# Patient and Healthcare Professional Priorities for a Mobile Phone Application for Patients With Peripheral Arterial Disease

**DOI:** 10.7759/cureus.33993

**Published:** 2023-01-20

**Authors:** Pavithira Sivagangan, Amy E Harwood, Philip W Stather

**Affiliations:** 1 Norwich Medical School, University of East Anglia, Norwich, GBR; 2 Centre for Sports, Exercise and Life Sciences, Coventry University, Coventry, GBR; 3 Vascular Surgery, Norfolk and Norwich University Hospitals NHS Foundation Trust, Norwich, GBR

**Keywords:** peripheral vascular surgery, digital health technology, home-based exercise, mobile apps (mhealth), peripheral arterial disease (pad)

## Abstract

Introduction

Supervised exercise therapy (SET) is the first-line treatment for the peripheral arterial disease (PAD), however, access and compliance are low. An alternative method of delivering this therapy is through mobile health applications, which can be more accessible and convenient for patients. The aim of this study is to evaluate patient, public and healthcare professional (HCP) priorities with regard to a dedicated mobile phone application to deliver remote SET.

Methods

Bespoke questionnaires were designed for patients and HCPs to assess app functionality and prioritisations for development. These were distributed through social media and the Norfolk and Norwich University Hospital.

Results

Functionality questionnaires were completed by 62 patients and 44 HCPs. Eighty-four per cent of patients wanted their therapy to be monitored by their vascular team with the majority (78%) interested in measuring walking distances. Most patients (76%) were interested in watching exercise videos. These views were shared by HCPs.

A communication platform was prioritised for messaging and pictures by the patient (74% and 68% respectively), but not so by HCPs (40%). Documenting other forms of physical activity and the use of wearable technology was less valuable to patients but favoured by HCPs (50%). The ability to interact with other users was not prioritised by either group.

Conclusion

Delivery of a mobile phone application to deliver health programmes for SET in patients with PAD is an acceptable method for patients and HCPs. This data will enable the next stages of mobile phone application development to be appropriately prioritised, focusing on building exercise videos, a communication platform and further walking tests.

## Introduction

Patients with symptomatic peripheral arterial disease (PAD) typically present with intermittent claudication (IC), which is discomfort or pain in the lower limbs induced by walking and relieved by rest. Over time, pain-free walking distance decreases, affecting the quality of life, due to a decline in functional status. Eventually, this can lead to the requirement of surgical interventions and/or amputations due to tissue loss (caused by gangrene and ulceration) [1], if left untreated. The UK National Vascular Registry reported over 3000 major lower limb amputations in 2020 [2], secondary to PAD, with a 7.0% overall rate of 30-day in-hospital death.

Guidelines from the National Institute for Health and Care Excellence (NICE), European Society of Cardiology, European Society for Vascular Surgery, American Heart Association and American College of Cardiology recommend that first-line treatment for IC is supervised exercise therapy (SET) [3-5]. This should be offered to all patients with IC for two hours per week for a period of three months, where they are encouraged to exercise to the point of maximal pain [3]. SET significantly improves pain-free and maximum walking distances and the quality of life of patients and is considered superior to home-based exercise therapy (HBET) [6,7]. Benefits with regards to modification of risk factors have also been proven, specifically blood pressure and cholesterol [8]. However, there is a deficiency in the provision of SET, primarily due to the lack of accessible units (linked with funding issues), reduced patient compliance and low adherence rates [9,10]. The lack of available units may also lead to longer travel distances with consequential financial implications [11], further increasing the barriers to participation. The Vascular Society of Great Britain and Ireland has recently undertaken a James Lind Alliance priority-setting exercise, which has identified the need to improve access and provision of exercise therapy and the need for optimisation of exercise prescriptions [12]. This indicates that there is an unmet gap in service delivery due to limited access and suboptimal exercise prescription.

An alternative mode of delivering exercise therapy that complies with the guidelines is required to provide optimum care for patients. A meta-analysis including 23 studies and 1309 participants revealed that with remote monitoring, HBET is equivalent to SET [13]. This highlights that HBET with remote monitoring, otherwise known as Virtual SET (VSET), has the potential to effectively deliver this alternative exercise therapy [14]. These platforms typically use wearable technology.

The use of virtual platforms during the COVID-19 pandemic and the recent upsurge of wearable technology to monitor activity and exercise has opened up new avenues for the delivery of remotely monitored exercise programmes. These platforms can not only help measure activity but also act as a motivational or behavioural change tool. A new concept that is becoming increasingly available is mobile health technology. The use of wearable activity monitors has been heavily researched in patients with IC as it is a simple, yet cost-effective way to quantify patients’ physical activity [15]. This tracking-based technology can promote patients to integrate exercise into their daily lives, making it accessible for those who are unable to travel to SET classes.

Mobile health technology has the additional benefit of providing personalised healthcare with health educational advice and exercise videos. These additional components above simple Global Positioning System (GPS) tracking, as used with activity monitors, may encourage behavioural change and promote adherence [16,17]. These applications can help deliver virtual SET as well as provide real-time feedback directly to the clinician and provide health education. This extensive supervision can help monitor compliance and progress and assist in highlighting deteriorating patients, who may require invasive procedures, minimising delayed presentations. At present four mobile health apps exist for patients with PAD, however, none have undergone testing in the UK and none are currently available for widespread use [18].

This study aims to use co-design and development methods for the development of a bespoke PAD app by getting valuable input from patients and clinicians on the usability, acceptability and feasibility of this method of exercise delivery, as part of an iterative development process.

## Materials and methods

Objectives/outcome measures

The primary outcome is to determine the patient, public and HCP opinions on the introduction and usability of a bespoke mobile phone application for PAD. Secondary outcomes are to determine patient, public and HCP opinions on development priorities for this type of mobile phone application.

App functionality assessment

Patient, public and HCP opinion on development priorities was acquired through a design and features questionnaire specific to PAD (one for patients and the public, and one for HCPs; Appendices 1 and 2). This 18-item questionnaire asks users to rate their interests in each topic from 1 (not interested at all) to 5 (very interested). The questionnaire was distributed nationwide through social media and advertising, and also directly to patients through outpatient clinics in the Norfolk and Norwich University Hospital (NNUH).

Study design

The patient and public questionnaire and HCP questionnaire were used to gather feedback on which features of the application were most important to patients and HCPs. This bespoke questionnaire was developed by PS and PWS based on a review of mHealth components identified in the literature, the SET class in NNUH, and was further developed in collaboration with the Vascular Society PAD specialist interest group. This group consists of experts in the field of exercise therapy including physiotherapists, exercise physiologists and vascular surgeons. The surveys were both distributed through social media, advertisements through the Circulation Foundation and vascular outpatient clinics. Online answers were collated through onlinesurveys.co.uk and exported into Excel. Questionnaires completed in the clinic were added to the Excel database.

Ethical approval

Ethical approval was granted by the University of East Anglia for the Faculty of Medicine and Health Sciences Research (application ID: ETH2122-0702).

The study commenced in July 2021 with data collection completed in April 2022.

Data security

Data were collected anonymously through onlinesurveys.co.uk with separate surveys for patients and HCPs. In NNUH, paper questionnaires were completed in the clinic. No personal, identifiable information was shared with developers or anyone outside of the direct clinical care team. All data were kept within the vascular research team in a locked cabinet in a locked office. The study data was only accessible to the study personnel.

Statistical analysis

Survey questions were scored on a Likert 1-5 scale, with results calculated as a percentage and/or mean, and results ranked for popularity as appropriate. Data for each item was reported in linear graphs and percentages to highlight the option shared by the majority. Data was split into both patient and HCP groups.

## Results

Patient functionality questionnaire

**Figure 1:**
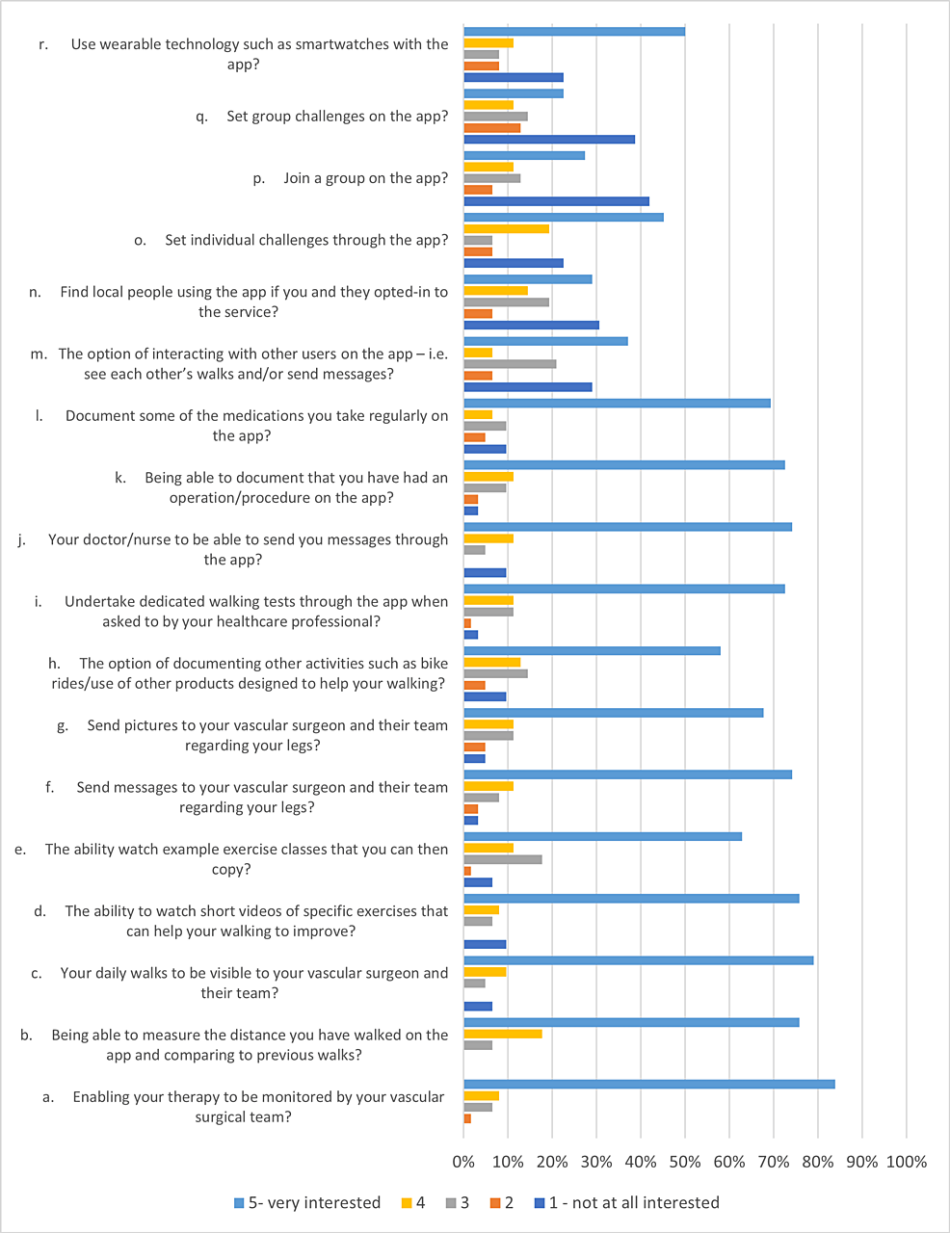
Bar graph showing patient and public responses to the app development survey.

The questionnaire was completed by 62 patients. All patients that completed the questionnaire had a smartphone. Eighty-four per cent wanted their therapy to be monitored by their vascular team, with no patients selecting that they were not at all interested in this notion (Figure 1). The majority of patients (78%) were also interested in being able to measure their walking distances with 79% also wanting their vascular team to be able to view their walks. Most patients (76%) were interested in being able to watch exercise videos and 62% were interested in being able to copy these exercise videos (Figure 1).

A communication platform was also valuable to patients with 74% being very interested in being able to send messages to the team (Figure 1) and 68% interested in sending pictures of their legs. Most patients (74%) were also keen on having the vascular team contact them through the application.

Documenting other forms of physical activity and the use of wearable technology was less valuable to patients (Figure 1). The ability to document medications and operations/procedures and carry out dedicated tests required by the vascular team was also of high interest to patients.

Interacting with other PAD users and carrying out group activities was less desirable with only 35% being very interested in conversing with other users (Figure 1). Additionally, 29% of patients preferred not to locate local people using the application and 41% and 38% were not interested in joining a group or setting group challenges, respectively. Also, setting individual challenges was desired by less than half of the patient respondents (45%).

Based on the summary statistics calculated (Table 1), the questions were ranked from most to least important for app development from a patient perspective, with 1 being the most important and 16 being the least important.

**Table 1:** Priority ranking for mobile phone application development by the patients and public by the mean score.

Question	Mean score
Therapy monitored by vascular team	4.75
Measuring walking distances and comparing with previous walks	4.69
Walks being visible to vascular team	4.55
Communication platform with vascular team (sending messages about legs)	4.50
Undertaking walking tests requested by vascular team	4.48
Documenting operations/procedures on the app	4.47
Vascular team sending messages through the app	4.40
Watching exercise videos to improve walking on the app	4.40
Sending pictures to the vascular team	4.32
To watch and then copy exercise classes	4.22
Documenting medication	4.21
Documenting other activities like bike rides	4.04
Use wearable technology such as Smartwatches with the app	3.85
Setting individual challenges and using wearable technology with the app	3.58
Interacting with other users	3.16
Finding local people using the app	3.04
Joining a group on the app	2.75
Setting group challenges	2.66

HCP questionnaire

The HCP survey received 44 responses and 95% agreed on the use of GPS to track walking distances, with 77% stating that both apps and websites are suitable platforms to view walking distances. Another suggested form was automated emails with walking logs.

**Figure 2:**
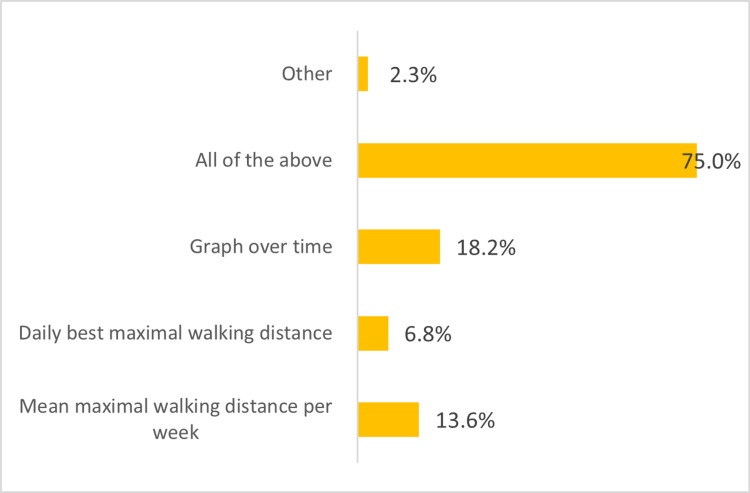
Bar graph showing healthcare practitioner preference for data output for patient monitoring.

There were multiple preferences for presenting patient data including a graph of walking distances over time, best daily maximal walking distance and mean distance per week. Another mode was suggested - an evolution of time taken to cover the walking distance (i.e. walking speed) (Figure 2).

**Figure 3:**
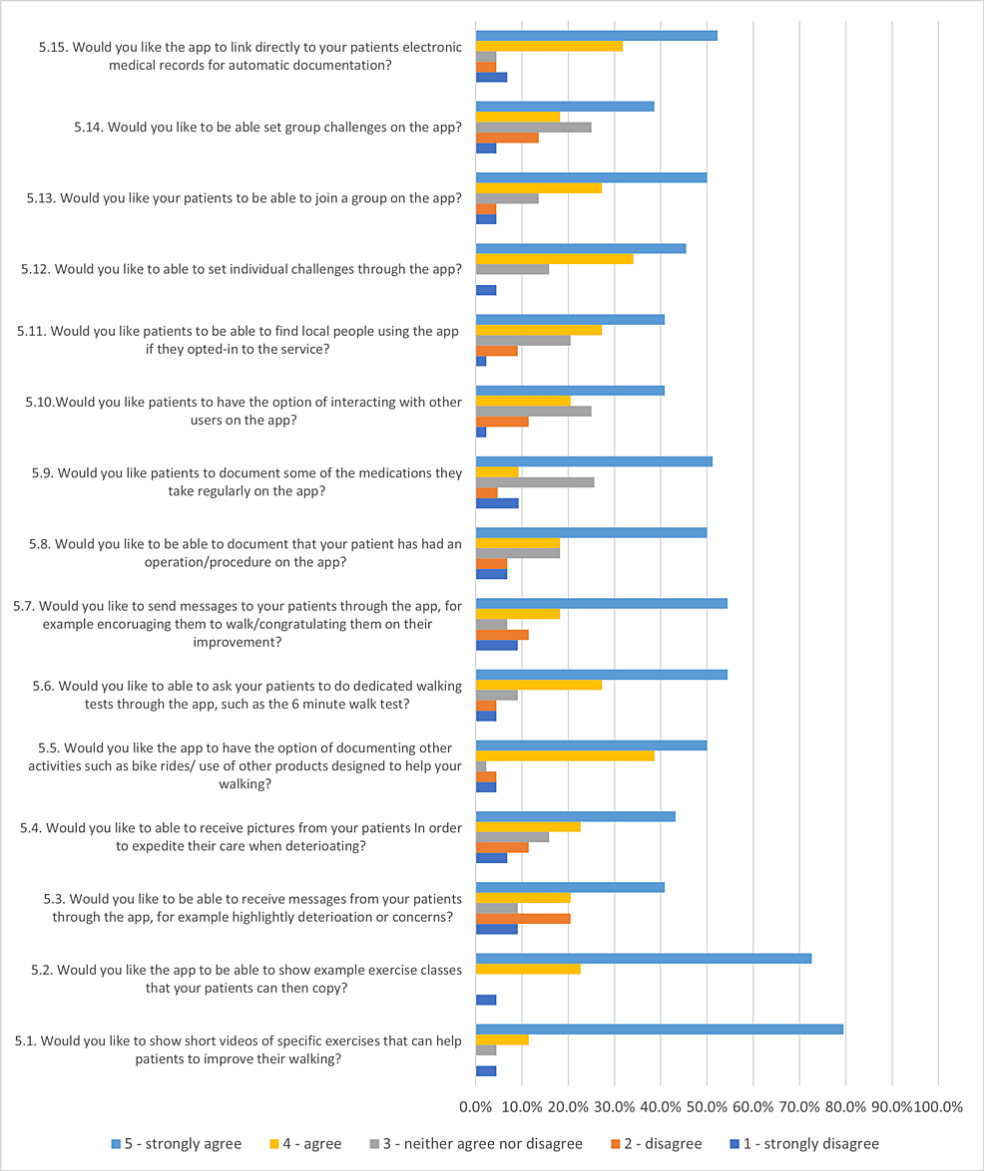
Bar graph showing patient and public responses to the app development survey.

Example exercise videos (Figure 3) and classes were well desired by HCPs with more than 70% selecting strongly agree on this feature. Most (55%) HCPs also supported the idea of asking patients to undertake walking tests such as the six-minute walk test to assist their therapy.

A communication forum with messaging (Figure 3) and picture services received mixed votes, but more than 40% of HCPs strongly agreed with this feature. The majority (55%) also strongly agreed on sending messages of encouragement to help motivate patients.

The ability to document other activities (Figure 3) and operations/procedures were again favoured as 50% selected strongly agree. The documentation of medications also followed at 51% as well as linking the app directly to electronic medical records at 52%.

Forty-one per cent of HCPs agreed with the idea of interacting with other users and locating local people. The idea of joining groups was desired at 50%. Setting individual and group challenges (Figure 3) ranked relatively low at 46% and 39% respectively. Based on the summary statistics (Table 2), the features that the HCP found to be most important to least important were ranked.

**Table 2:** Priority ranking for mobile phone application development by healthcare professionals by mean score.

Question	Mean score
Ability to monitor patients	4.61
Embed short videos in the app	4.59
Example exercise classes	4.25
Documenting other activities	4.23
Patients joining a group through the app	4.18
Linkage to electronic patient records	4.18
Set individual challenges	4.16
Undertake dedicated walking tests	3.98
Send messages to the patients	3.98
Patients interacting with other users	3.95
Document medications	3.86
Receive messages from patients	3.84
Document operations	3.80
Set group challenges	3.73
Receive pictures from patients	3.64

## Discussion

The introduction of mobile health technology could sustainably transform care delivery through continuous monitoring and direct communication between patients and HCP [19,20], all of which could reduce delayed presentations and minimise the need for outpatient appointments. This study evaluated whether the features of a mobile phone application would align with the features that patients, the public and HCP would like as part of a mobile health application to assist with the delivery of exercise therapy. From the patient perspective, the results revealed that enabling the vascular team to monitor and visualise the patients’ walk was the most desired feature, with a communication platform coming in second. Exercise videos and classes were deemed important by the HCPs. The addition of exercise classes and six-minute walk tests would transform the Walk-A-Cise application from a monitoring to a treatment platform.

Mobile phone-based health apps for PAD are novel but promising results were seen with another PAD-specific app ‘TrackPAD’ [21] as patients were satisfied with the functionality, aesthetics and amount of informative content. They have also been found to be acceptable to patients [22] and provide more personalise care [23].

To encourage uptake amongst this population, diverse perspectives have been obtained from patients and HCPs through questionnaires. This is important as patient and HCP preferences have been identified as critical in ensuring the needs of the target population are met [24]. The results identified that the idea of an application with supervision from the vascular team was well received by both patients and HCPs. In particular, the ability to measure walking distances and view previous walks was listed as a high-priority feature by patients. Previous research has shown that both patients and HCPs are equally interested in the ability to undertake dedicated walking tests in the community setting. Indeed, several studies in other clinical populations have demonstrated the validity and reproducibility of the six-minute walk test when used in the community [25-27].

A two-way communication platform was also thought to be desirable by both HCP and patients. This has several benefits including the ability to prompt patients to encourage routine use, as well as provide a direct line of communication for patients who have deteriorated, thus enabling a streamlined patient care pathway, leading to faster assessment and intervention where necessary. HCPs were highly keen on having exercise classes or videos that could be copied by their patients. However, the idea of interacting with other users was not particularly favoured by patients, which is contrary to the thoughts of HCPs. Indeed, many users of SET classes particularly enjoy the social interaction this entails.

The main limitation of this study was that the questionnaire was sent through established vascular routes such as the Circulation Foundation, and vascular-related Twitter sites, as well as vascular clinics. This is therefore prone to user bias. In addition, data regarding the patients and the public that undertook the questionnaires were not available such as age, gender and medical history, therefore we cannot be certain that all participants had PAD. Finally, not all patients have access to a smartphone, although smartphone uptake among the elderly is increasing on an annual basis.

## Conclusions

Delivery of a mHealth programme for SET in patients with PAD is an acceptable method for patients, the public and HCPs. This data will enable the next stages of mobile phone application development to be appropriately prioritised, focusing on building exercise videos, a communication platform and further walking tests. Further development and implementation of a mobile phone application developed specifically for patients with PAD opens the possibility of increasing remote consultations and earlier identification of deteriorating patients.

## Appendices

Appendix 1 - Patient and public involvement survey

Mobile App for Peripheral Arterial Disease Questionnaire

Dear Sir/Madam,

We are currently developing a mobile phone application for adults with peripheral arterial disease and would like to get feedback from both patients and healthcare professionals regarding the design and features of the app. It is primarily intended to improve home-based exercise therapy, monitoring and communication between the patient and the vascular clinical team. We would be very grateful if you could spare 10 minutes to answer the questions below which will help develop an app that both people with peripheral arterial disease and healthcare professionals can benefit from. By ticking the box below, you indicate that you consent to participate in this survey.

I Agree

1. Do you have a mobile phone that can access the Internet and use applications?

  Yes

  No

2. For the following questions please could you rate how interested you would be in the future below, with one being not interested at all, and five being very interested:

a. Enabling your therapy to be monitored by your vascular surgical team?

  1 - not at all interested

  2

  3

  4

  5 - very interested

b. Being able to measure the distance you have walked on the app and comparing to previous walks?

  1 - not at all interested

  2

  3

  4

  5 - very interested

c. Your daily walks to be visible to your vascular surgeon and their team?

  1 - not at all interested

  2

  3

  4

  5 - very interested

d. The ability to watch short videos of specific exercises that can help your walking to improve?

  1 - not at all interested

  2

  3

  4

  5 - very interested

e. The ability to watch example exercise classes that you can then copy?

  1 - not at all interested

  2

  3

  4

  5 - very interested

f. Send messages to your vascular surgeon and their team regarding your legs?

  1 - not at all interested

  2

  3

  4

  5 - very interested

g. Send pictures to your vascular surgeon and their team regarding your legs?

  1 - not at all interested

  2

  3

  4

  5 - very interested

h. The option of documenting other activities such as bike rides/use of other products designed to help your walking?

  1 - not at all interested

  2

  3

  4

  5 - very interested

i. Undertake dedicated walking tests through the app when asked to by your healthcare professional?

  1 - not at all interested

  2

  3

  4

  5 - very interested

j. Your doctor/nurse to be able to send you messages through the app?

  1 - not at all interested

  2

  3

  4

  5 - very interested

k. Being able to document that you have had an operation/procedure on the app?

  1 - not at all interested

  2

  3

  4

  5 - very interested

l. Document some of the medications you take regularly on the app?

  1 - not at all interested

  2

  3

  4

  5 - very interested

m. The option of interacting with other users on the app - i.e. see each other’s walks and/or send messages?

  1 - not at all interested

  2

  3

  4

  5 - very interested

n. Find local people using the app if you and they opted-in to the service?

  1 - not at all interested

  2

  3

  4

  5 - very interested

o. Set individual challenges through the app?

  1 - not at all interested

  2

  3

  4

  5 - very interested

p. Join a group on the app?

  1 - not at all interested

  2

  3

  4

  5 - very interested

q. Set group challenges on the app?

  1 - not at all interested

  2

  3

  4

  5 - very interested

r. Use wearable technology such as smartwatches with the app?

  1 - not at all interested

  2

  3

  4

  5 - very interested

3. If you would be interested in more about the development of this app and helping our research please leave your contact details below:

Appendix 2 - Healthcare practitioner survey

Dear colleague,

We are currently developing a mobile phone app for patients with peripheral arterial disease, and we would like to get feedback from both patients and healthcare professionals regarding the design and features of the app. It is primarily intended to improve exercise therapy and communication between the patient and the vascular team. We would be very grateful if you could spare 5 minutes to answer the questions below which will help develop an app that boot patients and healthcare professionals can benefit from. This survey is designed to access what a healthcare professional would like, NOT what you feel your patients would like. (Please answer the communication questions as you or a member of your team.) By ticking the box below, you agree to participate in this survey.

  I agree

1.

a. Would you or your team like to be able to monitor your patients’ walking distance over time using GPS tracking through a mobile phone app?

  Yes

  No

  Other

b. If you selected other, please specify:

2. 

a.  How would you like to be able to view your patients' walking distance?

  Website

  App

  Both

  Other

b.  If you selected other, please specify:

3.

a. How would you like your patients’ data to be presented?

  Mean maximal walking distance per week daily

  Daily best maximal walking distance

  Graph over time

  All of the above

  Other

b. If you selected other, please specify:

4. For the following questions please could you rate how interested you would be in the feature below with one being strongly disagree and 5 being strongly agree unless stated otherwise:

a. Would you like to show short videos of specific exercises that can help patients to improve their walking?

  1 - strongly disagree

  2 - disagree

  3 - neither agree nor disagree

  4 - agree

  5 - strongly agree

b. Would you like the app to be able to show example exercise classes that your patients can then copy?

  1 - strongly disagree

  2 - disagree

  3 - neither agree nor disagree

  4 - agree

  5 - strongly agree

c. Would you like to be able to receive messages from your patients through the app for example highlighting deterioration or concerns?

  1 - strongly disagree

  2 - disagree

  3 - neither agree nor disagree

  4 - agree

  5 - strongly agree

d. Would you like to be able to receive pictures from your patient in order to expedite their care when deteriorating?

  1 - strongly disagree

  2 - disagree

  3 - neither agree nor disagree

  4 - agree

  5 - strongly agree

e. Would you like the app to have the option of documenting other activities such as bike rides or the use of other products designed to help your walking?

  1 - strongly disagree

  2 - disagree

  3 - neither agree nor disagree

  4 - agree

  5 - strongly agree

f.  Would you like to be able to ask your patience to do dedicated walking tests through the app such as the six-minute walk test?

  1 - strongly disagree

  2 - disagree

  3 - neither agree nor disagree

  4 - agree

  5 - strongly agree

g. Would you like to be able to send messages to your patients through the app for example encouraging them to walk/congratulating them on their improvement?

  1 - strongly disagree

  2 - disagree

  3 - neither agree nor disagree

  4 - agree

  5 - strongly agree

h. Would you like to be able to document that your patient has had an operation procedure on the app?

  1 - strongly disagree

  2 - disagree

  3 - neither agree nor disagree

  4 - agree

  5 - strongly agree

i. Would you like patience to document some of the medications they take regularly on the app?

  1 - strongly disagree

  2 - disagree

  3 - neither agree nor disagree

  4 - agree

  5 - strongly agree

j. Would you like patience to have the option of interacting with other users on the app?

  1 - strongly disagree

  2 - disagree

  3 - neither agree nor disagree

  4 - agree

  5 - strongly agree

k. Would you like patience to be able to find local people using the app if they opted-in to the service?

  1 - strongly disagree

  2 - disagree

  3 - neither agree nor disagree

  4 - agree

  5 - strongly agree

l. Would you like to be able to set individual challenges through the app?

  1 - strongly disagree

  2 - disagree

  3 - neither agree nor disagree

  4 - agree

  5 - strongly agree

m. Would you like your patients to be able to join a group on the app?

  1 - strongly disagree

  2 - disagree

  3 - neither agree nor disagree

  4 - agree

  5 - strongly agree

n. Would you like to be able to set group challenges on the app?

  1 - strongly disagree

  2 - disagree

  3 - neither agree nor disagree

  4 - agree

  5 - strongly agree

o. Would you like the app to link directly to your patients’ electronic medical records for automatic documentation?

  1 - strongly disagree

  2 - disagree

  3 - neither agree nor disagree

  4 - agree

  5 - strongly agree

5. If you would be interested in more about the development of this app and helping our research please leave your contact details in the box below.
